# Distance-amplified power-law distributions better characterize human long-distance travel

**DOI:** 10.1038/s41598-026-37165-y

**Published:** 2026-01-31

**Authors:** Gregor Bankhamer, Huiran Liu, Souneil Park, Robert Elsässer, Stefan Schmid

**Affiliations:** 1 Electrical Engineering and Computer Science, Technische Universität Berlin, 10587 Berlin, Germany; 2 Telefónica R&D, Telefónica, 08019 Barcelona, Spain; 3 Department of Computer Science, University of Salzburg, 5020 Salzburg, Austria

**Keywords:** Mathematics and computing, Physics

## Abstract

Human mobility patterns have been the subject of research for many decades. Understanding long-distance trips is critical in our globalized world, for example, to model the spread of diseases. Traditional models generally assume that trip lengths follow a power-law distribution. We analyze over one million long-distance trips using three datasets: two survey-based (from Germany and the U.S.) and one from mobile network data in the U.K. We find that the observed trip length distributions deviate from typical power-law behavior, motivating a new approach. In addition, we examine COVID-19 spreading patterns in Germany and identify mobility dynamics that traditional power-law models fail to capture. To address these limitations, we introduce a model that extends the power-law framework by amplifying long-distance trips – based on the intuition that once a journey exceeds a certain length, the remaining distance is also likely to be substantial. Our experiments underscore the need for advanced models of long-distance travel and demonstrate that distance amplification can enhance the accuracy of conventional models.

## Introduction

The basic laws governing human motion have fascinated researchers for many decades^1,2^. Indeed, understanding human mobility is important, and mobility models have many applications, including $\text {CO}_2$ emission analysis, infrastructure planning, resource optimization, and crisis response^3–10^. In particular, the question of how human mobility affects disease spread received much attention in the past^11–18^.

Traditional human mobility models usually revolve around power-law distributions or truncated variants of these distributions^2,19–25^. That is, trip lengths are typically heavy-tailed. Our work is motivated by empirical observations drawn from three large datasets from Germany^26^, the United States^27^, and the United Kingdom, which show that while existing human mobility models relying on power-law distributions describe short- and medium-distance travel well, long-distance travel appears to be fundamentally different in nature. Understanding such long-distance travel is essential, as it serves as a known vector for the global spread of diseases^28,29^ and is becoming more frequent. Data from Deutsche Bahn AG^30^ shows that the number of long-distance train passengers rose from around 131 million in 2013 to 151 million in 2019.

**Fig. 1:**
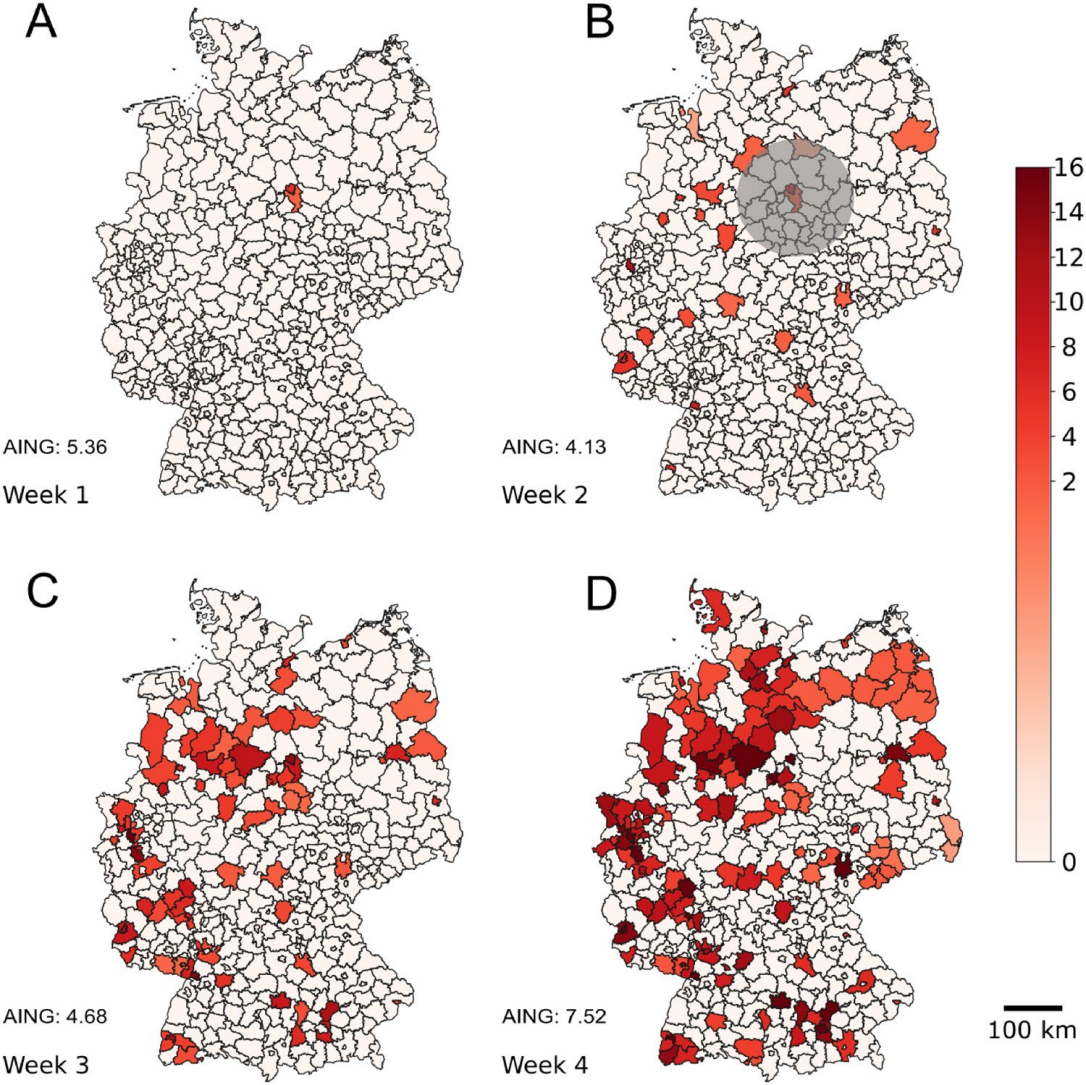
The disease first spreads at distant places (and then spreads locally). The figure is based on the average 7-day incidence rates of COVID-19 cases in all German counties (*Landkreise* and *Stadtkreise*), according to data from the Robert Koch Institute (RKI)^31,39–42^. To highlight the spread of the COVID-19 virus, we color a county red if the increase in incidence compared to the previous week is at least 20% for 3 consecutive weeks. In these cases, the county is colored red in all the weeks of such a sequence. Intuitively, this should only highlight counties in which there is currently an outbreak and allows us to observe newly created COVID-19 hotspots as the virus spreads in Germany. The intensity of the color depends on the magnitude of the 7-day incidence averaged over all days in that week. In Week 0 (06/16/2021 to 06/22/2021), more than 85% of the counties have incidences below 10 cases per 100,000 population, and no county would be colored. In all but one county with an incidence above 10 in Week 0, these values fall below 10 within the next 3 weeks. (**A**) shows the week from 06/23/2021 to 06/29/2021, (**B**) from 06/30/2021 to 07/06/2021, (**C**) from 07/07/2021 to 07/13/2021, and (**D**) from 07/14/2021 to 07/20/2021. Assuming that the overall distribution of trip lengths follows a power law (with exponent 2.13 as approximated for German mobility data^20^), at least 97% of all trips that start in the colored counties in Week 1 and end outside these counties should stop in the highlighted circular area (in gray) in (**B**). However, the virus instead evolves in geographically more distant regions and spreads only locally in later weeks. AING denotes the average incidence rate (per 100,000 inhabitants) in Germany in the corresponding weeks.

To provide some intuition as to why long-distance travel is important and how it relates to disease spread, we show in Fig. 1 how COVID-19 spread in the different German counties for four exemplary consecutive weeks at the beginning of the fourth wave^31^ (see caption for details). We observe that the virus first begins to grow in geographically distant areas and only later spreads around the initial sites. Similar observations of this spatio-temporal pattern of COVID-19 spread have been made in the literature before^15,16,32–35^. Note that this does not prove (nor is it the case) that the virus is transmitted from the location colored in Week 1 to all locations colored in Week 2 (cf. Fig. 1). However, the spreading behavior observed in Fig. 1 cannot be explained by traditional power-law based processes^19,22,36–38^. While in Fig. 1 the behavior of the epidemic is based on several phenomena occurring simultaneously (i.e., the Alpha (B.1.1.7) variant decreases in almost all counties while the Delta (B.1.617.2) variant increases). In Supplementary Discussion 1 we further investigate the spread of a single variant (B.1.617.2) in its early days in Germany. Note that the behavior observed w.r.t. the variant B.1.617.2 also seems to indicate that especially long-distance travel does not follow a power-law distribution. More specifically, if the distances followed a power-law distribution with an exponent around 2 or more, then the resulting effective distances (as defined in the seminal paper by Brockmann and Helbing^18^) would lead to a a different spreading behavior.

Above observations motivate us to present a new model that we show to capture long-distance travel well. Our model accounts for our empirical observation that people travel to distant places more frequently than a power-law distribution would suggest. The model is related to existing biological models, in particular to Lévy flight processes^36^; however, in contrast to existing models, we model long-distance trips with an amplification process. Concretely, our model amplifies a power-law with a second distribution, resulting in a process we call an amplified power-law. The corresponding distribution of the distances is called amplified power-law distribution. Additionally, we enhance our model with a novel dynamic truncation method that accounts for trip distance limits naturally imposed by national borders and can be applied to existing models.

Other studies have observed the limitations of basic power-law models in context of human mobility^25,43–47^. In this work, we focus on emphasizing the shortcomings of power-law–based approaches specifically in the context of long-range mobility. Our baseline model remains deliberately simple and can be trained given just a set of empirically observed trip distances. In contrast, many contemporary approaches rely on additional inputs-such as spatial structure, population density estimates, or temporal information- to capture human movement dynamics more accurately^44–54^. Our model also differs from the Lévy flights model often considered in early literature, which has been shown to generally describe well human activities such as travel^19,22,36,37,55^, and animal foraging^23,56,57^. In that model, movement distances usually follow a (truncated) power-law.

For our empirical considerations, we draw on three large human mobility datasets. Two are based on surveys (by mail and phone) in Germany and the United States consisting of 892,627 and 923,572 reported trips, respectively. They include additional information on transport modes and report actual distances, thus avoiding the biases of check-in-based methods and complementing existing studies based on cellular phone data or indirect surveys^17,20,58,59^. To enhance coverage and generalizability, we also include a third dataset from the United Kingdom, consisting of 1,001,069 trips inferred from the movement of mobile devices between cellular antennas.

## Results

We first define the notion of long-distance trips, and on this occasion present our datasets. We then present our empirical observations and introduce our distance amplified model.

### Long-distance trips

Our empirical analysis*d*raws upon three datasets, two of which are survey-based and report transport modes, while the third is passively derived from mobile network data. Each dataset is a collection of trips. A trip corresponds to a singular travel event and is associated with a distance value. Repeated trips of same length (e.g., trips between the same origin-destination pair) are recorded as separate observations and therefore appear multiple times in the dataset. We define long-distance travel based on dataset-specific criteria as follows:

- *Mobility in Germany (MID) 2017*^26^. Long-distance trips are defined as those taken using transport modes typically associated with travel over greater distances. These include car (*Fahrer*), long-distance train (*Fernzug*), long-distance bus (*Fernlinienbus*), coach (*Reisebus*), or airplane (*Flugzeug*). We focused our experiments on trips exceeding 100 km in length, leaving 11,008 trips for analysis.
- *American National Household Travel (NHTS) 2017*^27^. In this dataset, long-distance travel includes trips made by car, intercity bus (e.g., *Greyhound*, *Megabus*), airplane, or boat. Given the larger geographic scale of the U.S., we restrict our analysis to trips between 300 km and 5,000 km in length, with the upper bound reflecting the approximate maximum domestic air distance (excluding Alaska and Hawaii). Limiting the range of analyzed distributions is common in statistical modeling^60^, especially when fitting heavy-tailed distributions such as power laws. The remaining dataset consists of 2,853 trips.
- *U.K. Mobile Network Operator Data (MNO) 2022.* This passively collected dataset estimates trip distances based on the locations of the cell towers to which a mobile device connects at trip origin and destination. As transport modes are not recorded, we classify all trips longer than 100 km as long-distance. To focus on mainland U.K., we exclude trips exceeding 900 km. The final dataset comprises 1,000,717 trips.

### Short- and long-distance travel differs in nature

**Fig. 2:**
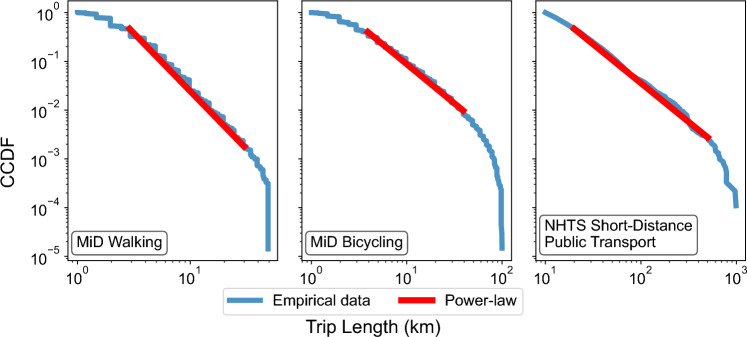
Short- and medium-distance trips exhibit power-law properties. The figure shows the distance distributions of short- and medium-distance trips from Germany (MiD) and the U.S. (NHTS) for different transport modes (i.e., walking, driving, and short-distance public transport). We compare these distributions with a power-law model $F(x) \propto (\frac{1}{x})^{\alpha -1}$. In all cases, we observe a linear trend and obtain a sufficiently high $\alpha$ value (at least 2), supporting the power-law assumption. From left to right, the panels show log–log scaled CCDFs for: walking trips in the MiD dataset; bicycle trips in the MiD dataset; and trips via short-distance public transport modes in the NHTS dataset.

When analyzing trips made by various transport modes suited for short-range travel, we observe characteristic power-law patterns. Specifically, we examine the complementary cumulative distribution functions (CCDFs) for walking, cycling, and short-distance public transport trips (e.g. school bus, public bus, subway and taxi), as shown in Fig. 2.

Each CCDF is compared to a reference power-law trend (shown as a red line), defined by the function $F(x) \propto (\frac{1}{x})^{\alpha -1}$ for a parameter $\alpha> 1$. On a log–log scale, such a power-law CCDF appears as a straight line, with a slope determined by the value of $\alpha$. We find that power-law trends with parameters $\alpha = 3.38$, $\alpha = 2.61$ and $\alpha = 1.59$, provide a close fit to large segments of the empirical distributions for the 69,987 walking trips, 67,639 cycling trips, and 9,130 short-range public transport trips, respectively.

### Long-distance trips are different

**Fig. 3:**
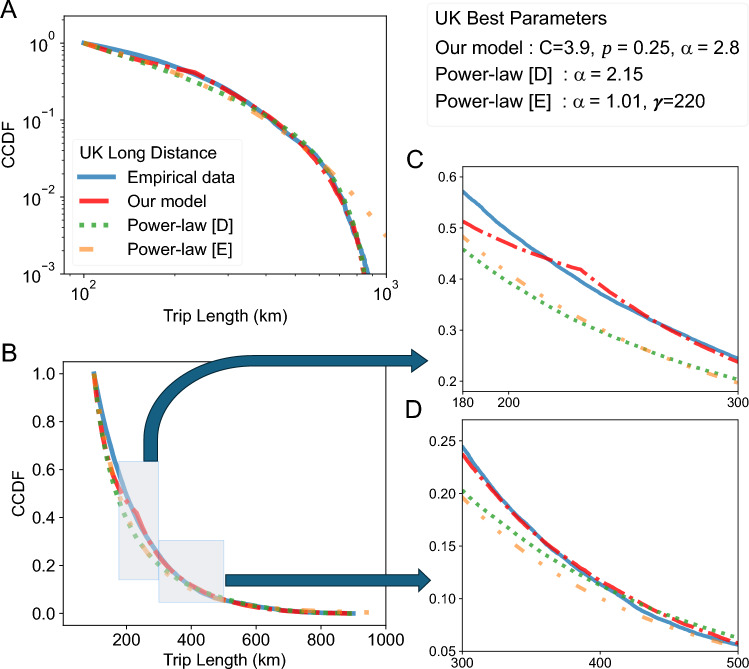
Long-distance travel in the U.K. exhibits non power-law properties. The distribution of long-distance trips in the MNO dataset compared to best-fit variants of several models, optimized based on the symmetric mean absolute percentage error (sMAPE)^61^. (**A**) and (**B**) show the distribution of long-distance trips with and without a log–log scale, respectively. In (**A**), the vertical axis is truncated at $10^{-3}$. (**C**) and (**D**) provide magnified views of the unscaled distribution, focusing on the distance ranges 180-300 km and 300-500 km, respectively. Unlike the short- and medium-distance trip distributions in Fig. 2, we cannot observe the linear trend on a log–log scale typical of a power-law.

**Fig. 4:**
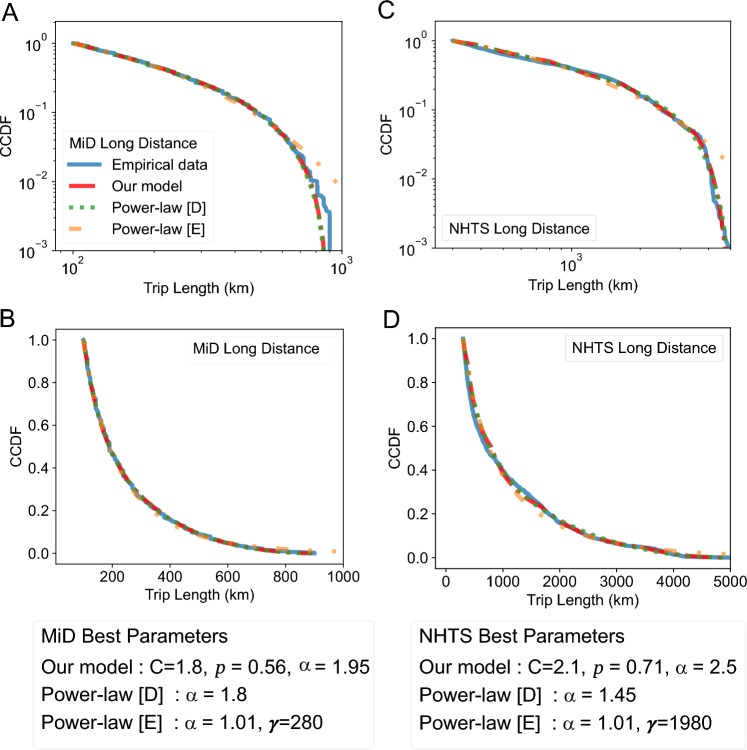
Long-distance travel in Germany and the U.S. follows similar trends. The figure shows the CCDF of long-distance trips in the MiD and NHTS datasets, compared to three models that were optimized by minimizing the sMAPE^61^. (**A**) and (**B**), show the MiD long-distance data (in blue) alongside the three fitted models. (**A**) is log–log scaled and has its vertical axis truncated at $10^{-3}$ probability mass, while (**B**) uses linear scales. Similarly, (**C**) and (**D**) present the corresponding analysis for the NHTS dataset. As with the MNO data, we cannot observe the clear linear trend of a power-law on a log–log scale.

A different pattern emerges when examining the CCDFs of long-distance trips across our three datasets. We focus on analyzing the distribution of trips derived from the movement of mobile devices between cellular antennas in the U.K. (see Fig. 3), as it represents our largest dataset. However, most observations also apply to the German and U.S datasets shown later in Fig. 4.

We first observe that the empirical distribution (in blue) looks quite different from the one for shorter trips in Fig. 2, as the typical straight trend of a power-law distribution is missing in the log-log plots (see Figs. 3A, 4A and C). Additionally, in the context of COVID-19, long-distance infections often occur at distances farther than a simple power law would suggest (see Fig. 1). If the distribution of all trips combined (short, medium, and long-distance) were to follow a power law with exponent $\alpha> 2$ (as approximated in related studies of German mobility data^20^), then the pattern of disease spread should differ from that observed in Fig. 1. In such a power-law model, in the second week, 97% of the trips starting from the two colored counties in Week 1 and ending outside of those counties will remain within the circular area (in gray). Accordingly, we would expect more infections in this circular area. This indicates that another model may be required to describe such trips. In our experiments, we compared the datasets to three different models all of which can be tuned given just a set of trip distances.

- *Power-law with Exponential Truncation (short: Power-law [E]).* This model is frequently considered in related work on human and animal mobility^2,23–25,55^. It’s density function *h* is proportional to $h(x) \propto x^{-\alpha } \cdot \exp (-x/\gamma )$ for a power-law exponent $\alpha$ and an exponential decay parameter $\gamma$.
- *Power-law with Dynamic Truncation (Power-law [D]).* This model consists of a simple power-law trend with density $j(x) \propto x^{-\alpha }$ that is truncated using our newly introduced dynamic truncation scheme.
- *Distance Amplification Model (Our Model).* This model is also based on a power-law trend, which is then potentially amplified multiple times. Details are given in the next section.

We also considered another related model^45^, whose approximated form can be trained using trip distances alone. This approximation resembles an exponentially truncated power-law and yields a less accurate fit under our metrics, while the full model requires temporal information. For completeness, we include a comparison in Supplementary Fig. 3. All of the models discussed above provide a better fit to the data than the standard, non-truncated power-law, which can only capture linear trends on a log–log scale. However, even the well-established exponentially truncated power-law distribution has notable limitations. Typically, the CCDF of this distribution initially shows a linear trend in log–log space, before decreasing exponentially as trip distances approach $\gamma$. As we can observe in Figs. 3A and 4A,C this type of truncation is not sufficiently strong, resulting in an overestimation of the number of long-distance trips. When considering the power-law distribution employed with our dynamic truncation (described in the next section), depicted in green, we find an improved performance. However, as observed between 180 km and 300 km in the U.K. dataset (see Fig. 3C), this model also deviates significantly from the empirical data. It inherits the typical initial linear trend of a power-law in log–log space (see the green line between 100 and 300 km in Fig. 3A), which does not accurately reflect the observed data in this region.

This motivates the development of a new model tailored to long-distance trips. The red curves in Figs. 3 and 4 represent our proposed model, which better describes the empirical data. Especially in the CCDF of the MNO data, with distance ranges depicted in Fig. 3C and D, we observe substantial improvements. In the former range, our model captures an increased concentration of trips around the 230 km mark, leading to a steeper decline in the CCDF and thus a more faithful representation of the empirical distribution.

### A model for long-distance travel

To capture both the characteristics of short-distance trips typically considered in the literature and the distance-amplification process of long-distance trips revealed in this paper, we propose a product distribution to model trip distances. One of these distributions is a standard power-law distribution $\textrm{Pow}(\alpha )$ with parameter $\alpha> 1$ and density function $f(x) = (\alpha -1) \cdot x^{-\alpha }$. Distances $X \sim \textrm{Pow}(\alpha )$ drawn from this distribution are amplified by multiplication with factor *M*, which is drawn from the other distribution denoted by $\textrm{Amp}(C,p)$ with parameters $p \ge 0$ and $C> 1$.

Intuitively, if a fixed individual has reached a distance *k* during a long-distance trip (e.g., traveling by plane or train), this person is likely to continue this trip for a distance that depends on *k*. Our distribution $\textrm{Amp}(C,p)$ captures this behavior, allowing the trip to continue with probability *p* and increasing its length to $C \cdot k$. The distribution allows multiple such amplifications to occur. This can be motivated as follows: A person using, for example, a long-distance train must first travel to a nearby station where such trains operate. If this initial distance is substantial, the remaining train journey is likely to cover a long distance; otherwise, the effort to reach the station would not be justified. Therefore, we model the total trip distance as a scaled amplification of this initial distance. Additionally, we observe that for high-speed trains, stops are far apart from each other (usually more than 50 km, e.g., in Germany and France), which is not common for local or regional trains. In most cases, people using high-speed trains do not leave the train after one stop. Therefore, we believe that the distance traveled so far is a lower bound for the remaining distance of the trip for many trips. Our model implements this intuition via successive amplifications, each by factor *C*.

Formally, $\textrm{Amp}(C,p)$ is a discrete distribution defined on $\{C^i \,\text {|}\, i \in \mathbb {N}_0 \}$. For $p>0$, the value *M* is drawn from $\textrm{Amp}(C,p)$ with $\Pr [M = C^i] = p^i \cdot (1-p)$. In the special case of $p=0$, the distribution $\textrm{Amp}(C,p)$ is defined to always returns value 1. To compute the CCDF, note, conditioned on $M=C^{i}$, a trip distance of at least *x* is reached with $\Pr [X \ge x/C^{i}]$:

$$\begin{aligned} \Pr [X \cdot M \ge x]&= \sum _{i=0}^{\lfloor \log _C(x) \rfloor }\Pr [ X \ge x / C^i ~|~ M = C^i] \cdot \Pr [M = C^i] \\&=\sum _{i=0}^{\lfloor \log _C (x) \rfloor } \left( \frac{C^{i}}{x} \right) ^{\alpha - 1} \cdot p^i (1-p) = \frac{1-p}{x^{\alpha -1}} \sum _{i=0}^{\lfloor \log _C (x) \rfloor } \left( pC^{\alpha -1}\right) ^i \\&= \frac{1-p}{x^{\alpha -1}} \cdot \frac{1-\left( pC^{\alpha -1} \right) ^{\lfloor \log _C(x) \rfloor + 1}}{ 1-pC^{\alpha -1}}. \end{aligned}$$

An alternative perspective on our product distribution model is a process in which an initial distance drawn from a power-law distribution is amplified multiple times by a factor *C*, where the number of amplifications is given by a geometric distribution with parameter *p*. Due to the heavy-tailed properties, many trips generated by the initial power-law distribution lie close to 1. As these trips are then amplified, an increase in density around $C^{i}, i \in \mathbb {N}$, may be observed. This allows our model to vary the slope in the CCDF space and sometimes proved a better fit, as observed in Fig. 3C at around 230 km and (less pronounced) in the NHTS data at around 1800 km. Finally, we note that setting $p=0$ reduces the product distribution to a non-amplified power-law distribution $\textrm{Pow}(\alpha )$.

#### Dynamic truncation

A natural limiter of long-distance travel is the national border, as a high percentage of travelers in Germany, for example, travel within the country^30^. Our model captures these characteristics, and since our studies are limited to a specific bounded geographic region (in our case, Germany, the U.K. and the U.S.), where trips start and end in the same country, we propose a type of dynamic truncation that captures this behavior. Each trip *t* is limited by a maximum allowed distance $\tau _t$ If a simulated trip exceeds distance $\tau _t$, we repeatedly redraw a new distance from our amplified power-law distribution until it falls below $\tau _t$. The value of $\tau _t$ is dynamic as it is chosen individually for each trip. To determine $\tau _t$, each trip *t* is assigned a random starting position in the country, and then $\tau _t$ is set as the maximum distance from that location to any point along the country’s border. For simplicity, we approximated the area of Germany, the U.K. and the U.S. with a circular area of radius 500, 900 and 2500 km, respectively. We also assume that the starting positions of the trips within these areas are chosen uniformly at random. We believe that this type of truncation is more natural than the exponential decay used in related models such as the truncated power-law distribution. In fact, we believe that our truncation method may be of independent interest and also useful for other distributions. As shown in Figs. 3 and 4, the dynamically truncated power-law distribution offers a more accurate representation of long-distance travel than the conventional model with exponential truncation. In Table 1 we observe that dynamic truncation improves the symmetric mean average percentage error (sMAPE) of the best-fitting power-law distribution; however, it is still outperformed by our distance-amplified model.

**Table 1:** Dynamic truncation can improve modeling of intra-country travel. This table lists the obtained sMAPE values for the best-fitting models depicted in Figs. 3 and 4. Compared to the exponential truncation commonly used in related work, dynamic truncation enables power-law distributions to more accurately capture the empirical trip data. Our proposed model – the amplified power-law – also incorporates dynamic truncation and consistently achieves the lowest sMAPE across all our experiments.

	Power-law [E]	Power-law [D]	Our model
MiD	0.146	0.074	0.054
NHTS	0.357	0.143	0.121
MNO	0.311	0.133	0.095

## Discussion

Understanding long-distance travel is important because of its various applications but also because the percentage of long-distance trips has continued to increase in recent years^30^. Comparing our 2017 datasets with data collected in previous years, we observe that the percentage of trips longer than 100 km increased from a fraction of 1.32% in 2008 to 1.74% in 2017 in Germany (MiD) and from 1.64% in 2009 to 2.14% in 2017 in the U.S. (NHTS). Human mobility has often been described using the Lévy flight model, i.e., the distance traveled is modeled with a power-law distribution. However, when we analyze existing mobility data^20^, we find that the distribution of the aggregated data differs from a power-law, especially between 200 and 900 km. We also observed in Fig. 1, that COVID-19 infections occur within a short period of time at larger distances from each other, indicating that travel is not fully captured by Lévy flights. However, we also observe that while COVID-19 first appears in distant locations, influenza seems to spread quickly in local areas as well, see Supplementary Discussion 2 for more details. The spread of these modern diseases is fundamentally different from the spread of, e.g., the Black Death in the Middle Ages^62^. Using our recent mobility data, we can also distinguish between different types of travel. The analysis of these types leads to the observation that while the lengths of the trips traveled with most of the different transport modes indeed follow a power-law distribution, long-distance travel does not have this property. Thus, there is a need to explain and model this type of travel. One of the worst pandemics in human history, the Black Death appeared in Constantinople and several cities along the Mediterranean trade routes in 1347, and did not reach northern Europe (parts of Germany, Poland, Denmark, Sweden, and Norway) before the second half of 1349^63^. Even from the city of Toulouse, the disease took about two months to reach Paris^64^. Although the sophisticated countermeasures we experienced during the COVID-19 pandemic were unknown in the 14th century, the spread of the disease was extremely slow compared to modern times. This difference is likely due, at least in part, to the different patterns of human mobility in the 14th century compared to the 21st century, particularly with regard to long-distance travel.

### Additional distributions

**Fig. 5:**
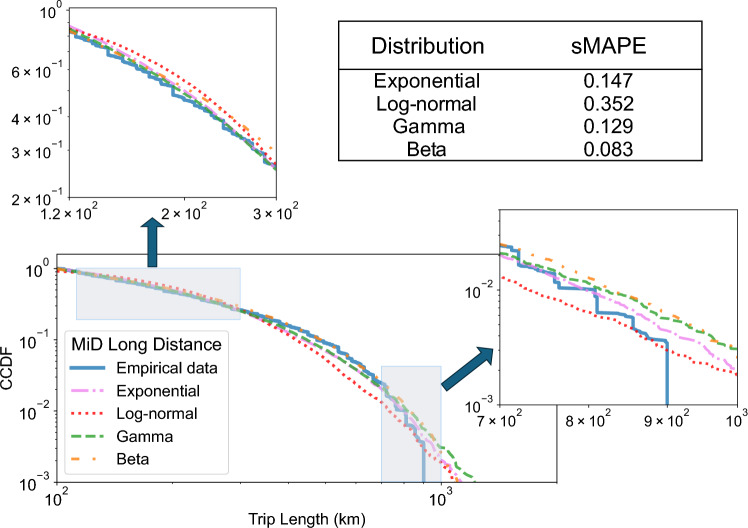
Other common distribution families also do not model long-distance data well. In this figure, we model the CCDF of the emperical long-distance data from the MiD dataset (cf. Fig. 4A) with the best-fitting exponential, log-normal, gamma and beta distribution CCDFs. The results are displayed in a log–log plot that is truncated at $10^{-3}$ on the vertical axis. We observe that none of the distributions manage to properly describe the tail of the distribution. Additionally, they tend to underestimate the number of trips with distance between 120 to 300 km. Out of all tested distributions, the beta distribution performs best with a sMAPE of 0.083.

We also investigated several non-powerlaw distribution families in order to find alternative simple models for long-distance travel. To that end, we considered the German MiD long-distance dataset and tested a number of commonly used distribution families (gamma, beta, exponential, and log-normal). Although none of these distributions capture the tail well (see Fig. 5), we found that the beta distribution provides the best performance. However, it is a wide class of distributions that lacks intuitive connection to human mobility. A fit with the gamma distribution distribution was refuted in the context of animal travel for similar reasons^65^. Additionally, the observed sMAPE of 0.083 in case of the beta distribution, is still 54% higher than the error of our model and is also higher than the error of the power-law with dynamic truncation (see Table 1). It should be noted that this difference in error would be even more pronounced in favor of our model; however, we limit the sMAPE calculation to approximately 900 km, and thus do not penalize the distributions for exceeding the tail of the empirical data.

### Transport mode segmentation

We also considered various transport modes in isolation. For example, trips by plain in the NHTS dataset and train in the MiD dataset. For these trips with isolated transport modes, we observed the same curved shape as the mixed empirical data presented in Fig. 4. Trends observed in our datasets therefore do not appear to be caused by mixing transport modes. In fact, most long-distance trips reported in the NHTS and MiD surveys were dominated by 1-2 transport modes, for example, airplane travel in the U.S. dataset.

### Limitations

Our model only allows for minimal input related to the country in which it is applied – namely, only an approximate value for the country’s diameter. In contrast, more sophisticated models incorporate richer contextual data and achieve impressive results. These approaches take into account, for example, the population density distribution, inter-city distances or the density of points-of-interest (for example workplace opportunities)^44,49–51^. While a detailed discussion of such models falls outside the scope of this work, several studies have compared these complex models against each other^52–54^. Another limitation of our work lies in the datasets used. The MiD and NHTS datasets are survey-based and therefore susceptible to human error, such as misreporting or rounding of distance values. To mitigate these issues, we supplement our analysis with the MNO dataset, which is passively generated from mobile device usage. This dataset avoids the aforementioned disadvantages but introduces its own challenges: it lacks transport mode information, and the raw data-captured as movements between cellular antennas-must be processed to infer discrete trips with identifiable start and end points. Extended pauses in movement may cause a trip to split into two segments, with each segment being recorded separately. Additionally, only trips starting from the designated home antenna of each device are recorded. However, despite the limitations inherent to each dataset, our analysis consistently reveals a deviation from a power-law trend across all long-range data sources. In the following section, we describe each dataset in greater detail.

## Methods

Our empirical results are based on three mobility datasets. The Mobility in Germany survey (MiD)^26^, the American National Household Travel Survey (NHTS)^27^ and the U.K. mobile network operator (MNO) data. MiD is a comprehensive nationwide survey commissioned by the Federal Ministry for Digital and Transport (BMDV), covering the daily travel behavior of households in Germany. Conducted in 2002, 2008, and 2017, it is the largest household survey other than the official German microcensus. The study uses a mixed methodology combining computer-assisted telephone interviews, as well as online and mail surveys to avoid bias. Participants self-report trip length values for each of the trips performed. The study covers all persons living in Germany, regardless of age. The MiD 2017 dataset contains data from June 2016 to September 2017. After removing all trips with unspecified distance values, it contains 136,357 households, 316,361 persons, and 892,627 trips. Each participant was assigned a randomly selected day and asked to record the trips made on that day. If more than one mode was used during a trip, that trip was assigned a single transport mode in the dataset according to a predefined hierarchy among these different modes. The NHTS is a survey that collects data on travel behavior in the U.S. The NHTS 2017 dataset is the eighth in a series of surveys. Sponsored by the Federal Highway Administration (FHWA), the survey collects data on daily travel by all modes for all purposes. The 2017 NHTS data includes a sample of 129,112 U.S. households and 923,572 trips. It was conducted from March 31, 2016 to May 8, 2017, with travel dates beginning on April 19, 2016 and ending on April 25, 2017. The survey covers a wide range of topics related to household characteristics, vehicle ownership and attributes, individual demographic characteristics, daily travel behavior, and opinions and experiences related to travel. Trip lengths are self-reported by participating individuals. The MNO dataset comprises a sample of 1,001,069 trips, each covering a distance of at least 100 kilometers, collected throughout November 2022. The dataset records device handovers as users connect and disconnect from antennas based on connection quality, typically selecting an antenna in close proximity to the device. The origin and destination of the trips are determined as follows:

- *Origin Identification:* The home antenna for each device is estimated by examining diurnal patterns, in accordance with prior literature^66,67^. Devices that consistently connect to the same antenna between midnight (00:00 am) and 8:00 am for at least 14 days within the month are designated as having their home location at that antenna. Devices that do not meet this criterion are excluded from the dataset.
- *Destination Detection:* The endpoints of the trips are identified based on connections to non-home antennas. A 30 minute minimum time threshold is applied to the duration of these connections to filter out potentially unstable connections that may occur while mobile-phone users are in transit or in areas with weak connectivity. For each such visited non-home antenna, a trip with distance from the home antenna to that antenna is recorded.

### Evaluation

In order to test whether the empirical data follows a certain distribution or model, several methods are known in the literature, e.g. Kolmogorov Smirnoff, Kuiper or Anderson-Darling tests^68^. To evaluate the distributions in our case, we compare their error according to sMAPE, which is also frequently employed in related studies of human mobility^69–71^. That is, we compute the error between the CCDF $F_{\mathcal {P}}$ of a distribution with parameter set ${\mathcal {P}}$ and the target CCDF *G* of the real-world data as follows. Over a fixed set of sample points ${\mathcal {S}}$, we evaluate the expression $\frac{2}{|{\mathcal {S}}|} \sum _{s \in {\mathcal {S}}} \frac{|F_{\mathcal {P}}(s) - G(s)|}{F_{\mathcal {P}}(s) + G(s)}$. The division by $F_{\mathcal {P}}(s)+G(s)$ normalizes sample contributions and accentuates discrepancies in the distribution’s tail, which is particularly relevant for modeling long-distance travel. For this reason, we adopted the sMAPE as our evaluation measure. In our experiments, the set ${\mathcal {S}}$ consisted of 1000 uniformly spaced samples, starting at our minimum distance thresholds for long-range travel and ending at distances such that less than $10^{-3}$ trips remain in the datasets (matching the visible range of the empirical data in Figs. 3A and 4A,C). An exception is made in case of the MiD data, which beyond approximately 800km becomes noisy as it only contains 111 remaining trips. Specifically, samples were drawn in the ranges $[100~\text {km}, 869~\text {km}]$, $[100~\text {km}, 800~\text {km}]$ and $[300~\text {km}, 4892~\text {km}]$ for the MNO, MiD and NHTS data, respectively. To optimize the parameters of the models considered in this paper and obtain the best-fits, we performed grid searches and selected the parameters ${\mathcal {P}}$ that yielded the smallest sMAPE. For each such combination of parameters ${\mathcal {P}}$, we simulated 50,000 trips of the considered model, which we used to approximate the CCDF $F_{\mathcal {P}}$ and calculate the error as above.

## Supplementary Information

Supplementary Information.

## Data Availability

The empirical data used in Figs. 2 and 4 is the 2017 Mobility in Germany (MiD) data and the 2017 National Household Travel Survey (NHTS) data. The 2017 MiD data is available upon request from Mobility in Germany. The 2017 NHTS data is publicly accessible and can be downloaded from the American National Household Travel Survey website in .csv format. The MNO dataset from November 2022 is subject to strict privacy regulations and not publicly available. It was anonymized and aggregated before being shared with the authors. The COVID-19 data presented in Fig. 1 is publicly available and can be obtained from the Robert Koch Institute (RKI).

## Code availability

The simulations were written in python 3.11. The corresponding code, including additional information on the required packages, is publicly available on GitHub and archived on Zenodo: https://doi.org/10.5281/zenodo.18187240. All scripts are configured to reflect the experiments performed in this manuscript. The experiments leading to the CCDFs of the power-law properties for short-distance trips (Fig. 2) and the CCDFs of the models for long-distance travel (Figs. 3, Fig. 4 and Fig. 5) can be run in a jupyter notebook after placing the datasets as described by the included README file.

## References

[1] Barbosa, H. et al. Human mobility: Models and applications. *Phys. Rep.* **734**, 1–74 (2018).

[2] Gonzalez, M. C., Hidalgo, C. A. & Barabasi, A.-L. Understanding individual human mobility patterns. *Nature* **453**, 779–782 (2008). 18528393 10.1038/nature06958

[3] Vespignani, A. Complex networks: the fragility of interdependency. *Nature* **464**, 984–985 (2010). 20393545 10.1038/464984a

[4] Helbing, D., Farkas, I. & Vicsek, T. Simulating dynamical features of escape panic. *Nature* **407**, 487–490 (2000). 11028994 10.1038/35035023

[5] Rozenfeld, H. D. et al. Laws of population growth. *Proc. Natl. Acad. Sci.* **105**, 18702–18707 (2008). 19033186 10.1073/pnas.0807435105 PMC2596244

[6] Horner, M. W. & O’Kelly, M. E. Embedding economies of scale concepts for hub network design. *J. Transp. Geogr.* **9**, 255–265 (2001).

[7] Wang, P., Hunter, T., Bayen, A. M., Schechtner, K. & González, M. C. Understanding road usage patterns in urban areas. *Sci. Rep.* **2**, 1001 (2012). 23259045 10.1038/srep01001 PMC3526957

[8] Pappalardo, L., Pedreschi, D., Smoreda, Z. & Giannotti, F. Using big data to study the link between human mobility and socio-economic development. In *2015 IEEE International Conference on Big Data (Big Data)*, 871–878 (IEEE, 2015).

[9] Gabaix, X., Gopikrishnan, P., Plerou, V. & Stanley, H. E. A theory of power-law distributions in financial market fluctuations. *Nature* **423**, 267–270 (2003). 12748636 10.1038/nature01624

[10] Banister, D. Cities, mobility and climate change. *J. Transp. Geogr.* **19**, 1538–1546 (2011).

[11] Hufnagel, L., Brockmann, D. & Geisel, T. Forecast and control of epidemics in a globalized world. *Proc. Natl. Acad. Sci.* **101**, 15124–15129 (2004). 15477600 10.1073/pnas.0308344101 PMC524041

[12] Colizza, V., Barrat, A., Barthelemy, M., Valleron, A.-J. & Vespignani, A. Modeling the worldwide spread of pandemic influenza: baseline case and containment interventions. *PLoS Med.* **4**, e13 (2007). 17253899 10.1371/journal.pmed.0040013 PMC1779816

[13] Belik, V., Geisel, T. & Brockmann, D. Natural human mobility patterns and spatial spread of infectious diseases. *Phys. Rev. X* **1**, 011001 (2011).

[14] Kleinberg, J. The wireless epidemic. *Nature* **449**, 287–288 (2007). 17882205 10.1038/449287a

[15] Alessandretti, L. What human mobility data tell us about Covid-19 spread. *Nat. Rev. Phys.* **4**, 12–13 (2022). 34877474 10.1038/s42254-021-00407-1 PMC8641537

[16] Zhang, J. et al. The impact of relaxing interventions on human contact patterns and SARS-CoV-2 transmission in China. *Sci. Adv.* **7**, eabe2584 (2021). 33962957 10.1126/sciadv.abe2584 PMC8104862

[17] Chang, S. et al. Mobility network models of Covid-19 explain inequities and inform reopening. *Nature* **589**, 82–87 (2021). 33171481 10.1038/s41586-020-2923-3

[18] Brockmann, D. & Helbing, D. The hidden geometry of complex, network-driven contagion phenomena. *Science* **342**, 1337–1342 (2013). 24337289 10.1126/science.1245200

[19] Brockmann, D., Hufnagel, L. & Geisel, T. The scaling laws of human travel. *Nature* **439**, 462–465 (2006). 16437114 10.1038/nature04292

[20] Wilkerson, G. J., Khalili, R. & Schmid, S. Urban mobility scaling: Lessons from ’little data’. In *2014 Proceedings IEEE INFOCOM Workshops, Toronto, ON, Canada, April 27 - May 2, 2014*, 777–782 (IEEE, 2014).

[21] Song, C., Koren, T., Wang, P. & Barabási, A.-L. Modelling the scaling properties of human mobility. *Nat. Phys.* **6**, 818–823 (2010).

[22] Raichlen, D. A. et al. Evidence of Lévy walk foraging patterns in human hunter-gatherers. *Proc. Natl. Acad. Sci.* **111**, 728–733 (2014). 24367098 10.1073/pnas.1318616111 PMC3896191

[23] Viswanathan, G. M. et al. Optimizing the success of random searches. *Nature* **401**, 911–914 (1999). 10553906 10.1038/44831

[24] Alessandretti, L., Sapiezynski, P., Lehmann, S. & Baronchelli, A. Multi-scale spatio-temporal analysis of human mobility. *PLoS ONE* **12**, 1–17 (2017). 10.1371/journal.pone.0171686 PMC5310761 28199347

[25] Yan, X.-Y., Han, X.-P., Wang, B.-H. & Zhou, T. Diversity of individual mobility patterns and emergence of aggregated scaling laws. *Sci. Rep.* **3** (2013). 10.1038/srep02678 PMC3776193 24045416

[26] German Federal Ministry for Digital and Transport. Mobility in germany (MiD) survey. Data is maintained by the infas Instituted for Applied Social Sciences.

[27] U.S. Department of Transportation. National household travel survey (NHTS). Data collected by Westat.

[28] Schlosser, F. et al. Covid-19 lockdown induces disease-mitigating structural changes in mobility networks. *Proc. Natl. Acad. Sci.* **117**, 32883–32890 (2020). 33273120 10.1073/pnas.2012326117 PMC7776901

[29] Brownstein, J. S., Wolfe, C. J. & Mandl, K. D. Empirical evidence for the effect of airline travel on inter-regional influenza spread in the United States. *PLoS Med.* **3**, 1–10 (2006). 10.1371/journal.pmed.0030401 PMC1564183 16968115

[30] Deutsche Bahn AG. Deutsche bahn 2022 integrated report (2022). Online: https://ibir.deutschebahn.com/2022/fileadmin/pdf/db_ib22_e_web.pdf (Accessed 19 Jan 2026).

[31] Robert Koch Institute (RKI). Covid-19 7-day incidence numbers per county in Germany (2021). Query online:https://survstat.rki.de/Content/Query/Create.aspx (Accessed 19 Jan 2026).

[32] Castro, M. et al. Spatiotemporal pattern of Covid-19 spread in Brazil. *Science* **372**, 821–826 (2021). 33853971 10.1126/science.abh1558

[33] Silva, G. C. & Ribeiro, E. M. S. The impact of Brazil’s transport network on the spread of Covid-19. *Sci. Rep.* **13**, 2240 (2023). 36755064 10.1038/s41598-022-27139-1 PMC9906601

[34] Ali, Y., Sharma, A. &amp; Haque, M. M. Transportation and a pandemic: A case study of Covid-19 pandemic. *Integrated Risk of Pandemic: Covid-19 Impacts, Resilience and Recommendations* 283–305 (2020).

[35] Witzke, S., Danz, N., Baum, K. & Renard, B. Y. Mobility data improve forecasting of Covid-19 incidence trends using graph neural networks. In *6th International Workshop on Epidemiology meets Data Mining and Knowledge Discovery (epiDAMIK)* (2023).

[36] Mandelbrot, B. B. & Mandelbrot, B. B. *The Fractal Geometry of Nature*, Vol. 1 (WH freeman New York, 1982).

[37] Kleinberg, J. M. Navigation in a small world. *Nature* **406**, 845–845 (2000). 10972276 10.1038/35022643

[38] Edwards, A. M. et al. Revisiting Lévy flight search patterns of wandering albatrosses, bumblebees and deer. *Nature* **449**, 1044–1048 (2007). 17960243 10.1038/nature06199

[39] Robert Koch Insitut (RKI). Bericht zu Virusvarianten von SARS-CoV-2 in Deutschland - 26. Mai 2021 (2021). https://www.rki.de/DE/Themen/Infektionskrankheiten/Infektionskrankheiten-A-Z/C/COVID-19-Pandemie/DESH/Berichte-VOC-tab.html (Accessed 19 Jan 2026).

[40] Robert Koch Insitut (RKI). Bericht zu Virusvarianten von SARS-CoV-2 in Deutschland - 02. Juni 2021 (2021). https://www.rki.de/DE/Themen/Infektionskrankheiten/Infektionskrankheiten-A-Z/C/COVID-19-Pandemie/DESH/Berichte-VOC-tab.html (Accessed 19 Jan 2026).

[41] Robert Koch Insitut (RKI). Bericht zu Virusvarianten von SARS-CoV-2 in Deutschland - 09. Juni 2021 (2021). https://www.rki.de/DE/Themen/Infektionskrankheiten/Infektionskrankheiten-A-Z/C/COVID-19-Pandemie/DESH/Berichte-VOC-tab.html (Accessed 19 Jan 2026).

[42] Robert Koch Insitut (RKI). Bericht zu Virusvarianten von SARS-CoV-2 in Deutschland - 16. Juni 2021 (2021). https://www.rki.de/DE/Themen/Infektionskrankheiten/Infektionskrankheiten-A-Z/C/COVID-19-Pandemie/DESH/Berichte-VOC-tab.html (Accessed 19 Jan 2026).

[43] Jurdak, R. et al. Understanding human mobility from twitter. *PLoS ONE* **10**, 1–16 (2015). 10.1371/journal.pone.0131469 PMC4496063 26154597

[44] Noulas, A., Scellato, S., Lambiotte, R., Pontil, M. & Mascolo, C. A tale of many cities: Universal patterns in human urban mobility. *PLoS ONE* **7**, 1–10 (2012). 10.1371/journal.pone.0037027 PMC3362592 22666339

[45] Gallotti, R., Bazzani, A., Rambaldi, S. & Barthelemy, M. A stochastic model of randomly accelerated walkers for human mobility. *Nat. Commun.* **7** (2016). 10.1038/ncomms12600 PMC5013551 27573984

[46] Alessandretti, L., Aslak, U. & Lehmann, S. The scales of human mobility. *Nature* **587**, 402–407 (2020). 33208961 10.1038/s41586-020-2909-1

[47] Boucherie, L., Maier, B. F. & Lehmann, S. Decoupling geographical constraints from human mobility. *Nat. Hum. Behav.* (2025). 10.1038/s41562-025-02282-7 40890439

[48] Nicolaides, C., Cueto-Felgueroso, L., González, M. C. & Juanes, R. A metric of influential spreading during contagion dynamics through the air transportation network. *PLoS ONE* **7**, e40961 (2012). 22829902 10.1371/journal.pone.0040961 PMC3400590

[49] Cabanas-Tirapu, O., Danús, L., Moro, E., Sales-Pardo, M. & Guimerà, R. Human mobility is well described by closed-form gravity-like models learned automatically from data. *Nat. Commun.* **16** (2025). 10.1038/s41467-025-56495-5 PMC11794706 39904994

[50] Liu, E.-J. & Yan, X.-Y. A universal opportunity model for human mobility. *Sci. Rep.* **10**, 4657 (2020). 32170196 10.1038/s41598-020-61613-y PMC7070048

[51] Simini, F., Barlacchi, G., Luca, M. & Pappalardo, L. A deep gravity model for mobility flows generation. *Nat. Commun.* **12**, 6576 (2021). 34772925 10.1038/s41467-021-26752-4 PMC8589995

[52] Simini, F., González, M. C., Maritan, A. & Barabási, A.-L. A universal model for mobility and migration patterns. *Nature* **484**, 96–100 (2012). 22367540 10.1038/nature10856

[53] Chico, Q. C., Bright, J. & Hale, S. A. Diagnosing the performance of human mobility models at small spatial scales using volunteered geographical information. *R. Soc. Open Sci.* **6** (2019). 10.1098/rsos.191034 PMC6894559 31827843

[54] Litmeyer, M.-L., Gareis, P. & Hennemann, S. Comparing student mobility pattern models. *Eur. J. Geogr.* **14**, 21–34 (2023).

[55] Barthelemy, M. *The Structure and Dynamics of Cities: Urban Data Analysis and Theoretical Modeling* (Cambridge University Press, 2016).

[56] Sims, D. W. et al. Scaling laws of marine predator search behaviour. *Nature* **451**, 1098–1102 (2008). 18305542 10.1038/nature06518

[57] Viswanathan, G. M. et al. Lévy flight search patterns of wandering albatrosses. *Nature* **381**, 413–415 (1996). 10.1038/nature06199 17960243

[58] Noulas, A., Scellato, S., Lambiotte, R., Pontil, M. & Mascolo, C. A tale of many cities: universal patterns in human urban mobility. *PLoS ONE* **7**, e37027 (2012). 22666339 10.1371/journal.pone.0037027 PMC3362592

[59] Ajitesh, S. *et al.* Nowcasting temporal trends using indirect surveys. In *Annual AAAI Conference on Artificial Intelligence* (2024).

[60] Newman, M. E. J. Power laws, pareto distributions and zipf’s law. *Contemp. Phys.* **46**, 323–351 (2005).

[61] Makridakis, S. & Hibon, M. The m3-competition: results, conclusions and implications. *Int. J. Forecast.* **16**, 451–476 (2000).

[62] Bos, K. *et al.* A draft genome of Yersinia Pestis from victims of the Black Death. *Nature*. **478** (2011). 10.1038/nature10549 PMC3690193 21993626

[63] Langer, W. Geographic and temporal development of plagues. *Sci. Am.* **2**, 114–121 (1964).

[64] Barker, H. Laying the corpses to rest. grain, embargoes, and yersinia pestis in the black sea. *Speculum*. **96**, 97–126 (2021).

[65] Boyer, D., Miramontes, O. & Ramos-Fernández, G. Evidence for biological Lévy flights stands. arXiv preprint (2008).

[66] Bojic, I., Massaro, E., Belyi, A., Sobolevsky, S. &amp; Ratti, C. Choosing the right home location definition method for the given dataset. In *Social Informatics* (eds Liu, T.-Y. et al.), 194–208 (Springer International Publishing, 2015).

[67] Park, S., Oshan, T. M., El Ali, A. & Finamore, A. Are we breaking bubbles as we move? using a large sample to explore the relationship between urban mobility and segregation. *Comput. Environ. Urban Syst.* **86**, 101585 (2021).

[68] Clauset, A., Shalizi, C. R. & Newman, M. E. J. Power-law distributions in empirical data. *SIAM Rev.* **51**, 661–703 (2009).

[69] Zhao, K., Tarkoma, S., Liu, S. & Vo, H. T. Urban human mobility data mining: An overview. In *2016 IEEE International Conference on Big Data (IEEE BigData 2016), Washington DC, USA, December 5-8, 2016*, 1911–1920 (IEEE Computer Society, 2016).

[70] Li, W., Wang, Q., Liu, Y., Small, M. L. & Gao, J. A spatiotemporal decay model of human mobility when facing large-scale crises. *Proc. Natl. Acad. Sci.* **119** (2022). 10.1073/pnas.2203042119 PMC9388161 35939676

[71] Li, X. et al. Prediction of urban human mobility using large-scale taxi traces and its applications. *Front. Comp. Sci.* **6**, 111–121 (2012).

