# Supplementary material for: Distance-amplified power-law distributions better characterize human long-distance travel
Source: Sci Rep. 2026 Jan 31;16:4331. doi: 10.1038/s41598-026-37165-y (PMC12864742; doi:10.1038/s41598-026-37165-y)
Supplement: Supplementary file 1 — Supplementary Information. [file 41598_2026_37165_MOESM1_ESM.pdf]

Supplementary Information:

Distance-Amplified Power-law Distributions Better Characterize Human Long-Distance Travel

Gregor Bankhamer[1], Huiran Liu[1], Souneil Park[2]
Robert Elsässer[3], Stefan Schmid[1*]

[1]Electrical Engineering and Computer Science, Technische Universität Berlin, 10587 Berlin, Germany

[2]Telefónica R&D, Telefónica, 08019 Barcelona, Spain

[3]Department of Computer Science, University of Salzburg, 5020 Salzburg, Austria

[*]Corresponding author. Email: stefan.schmid@tu-berlin.de

Contents

Supplementary Discussion 1 2

Supplementary Figure 1 3

Supplementary Discussion 2 4

Supplementary Figure 2 5

Supplementary Figure 3 6

Supplementary Discussion 1

Spread of COVID-19 Delta. While **Fig. 1** in the main paper illustrates the spread of the epidemic based on several concurrent phenomena (i.e., the Alpha (B.1.1.7) variant decreases in almost all counties while the Delta (B.1.617.2) variant increases), in **Supplementary Fig. 1** we track variant B.1.617.2, specifically. Note that the behavior observed in this figure also seems to indicate that especially long-distance travel does not follow a power-law distribution. If the distances followed a power-law distribution with an exponent around 2 or more, then the resulting effective distances (as defined in the seminal paper by Brockmann and Helbing[1]) would lead to a concentration of the virus in Week 17 around the locations where the virus was confirmed in Weeks 14-15. Instead, we see that the virus is widely distributed throughout the country in Week 17 of 2021. It should be noted that, especially in the early days of B.1.617.2 in Germany, several people infected with this variant were exposed to the virus outside of Germany prior to the onset. Nevertheless, by Week 20 of 2021, the number of confirmed or suspected such cases was less than 19% of all Delta cases, and most transmissions occurred within Germany[2].

Note that the visualization in **Supplementary Fig. 1** only captures one possible explanation for the spread of the virus during Weeks 17-21 (and is probably incomplete), as the data published in the reports has certain limitations. First, on county level the RKI aggregated confirmed and suspected cases of the Delta variant over a 4-week period in each of its reports, and according to our communications with the RKI, no non-aggregated data is available. Second, due to certain delays in the sequencing process of the tests, B.1.617.2 cases were often confirmed several weeks after the virus was detected by PCR in the nasopharyngeal swab of the individual. To obtain the possible spread of the Delta variant during the considered weeks, we analyzed tables with the occurrences of this variant in the different federal states of Germany (i.e., Table 8 in the report of May 26[2], Table 9 in the report of June 2[3], and Table 10 in the reports of June 9, June 16, and June 23[4–6]), as well as the maps with the distribution of the Delta variant across the counties (see Figure 4 in each of the cited RKI reports[2–6]). The report published by the end of Week 20 of 2021 mentions a total of 262 B.1.617 confirmed or suspected cases. Of these, 49 reported possible exposure to the virus outside of Germany prior to the onset[2].

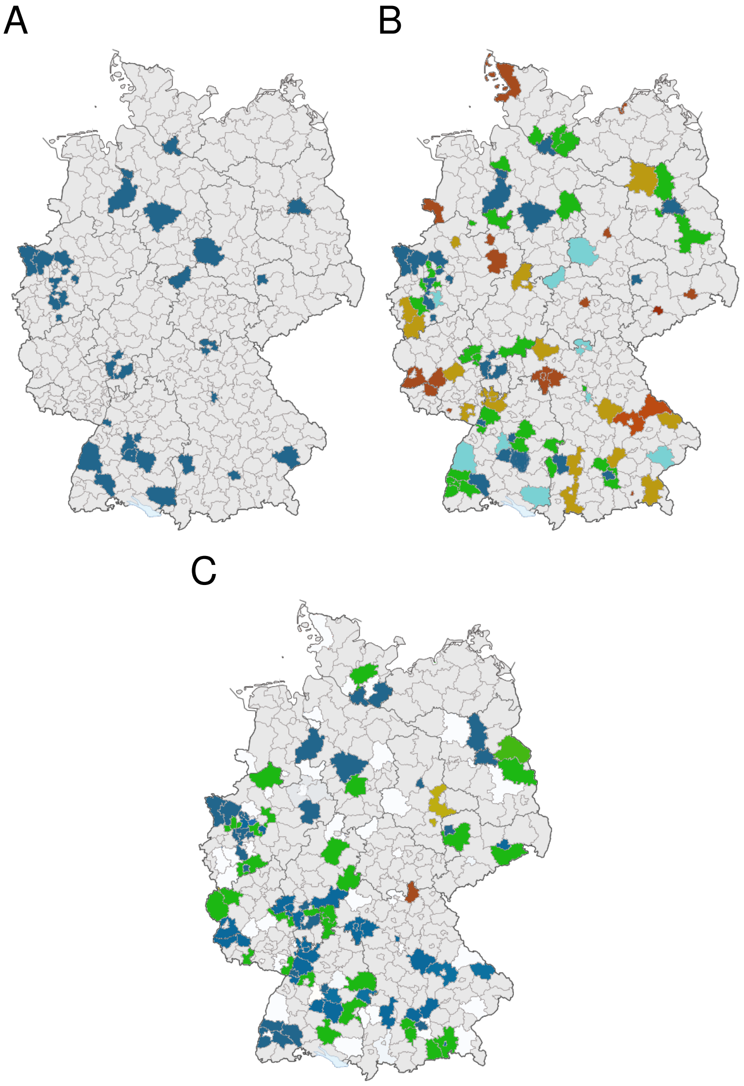

Supplementary Figure 1: Spread of the Delta variant in its early days in Germany. According to the weekly reports of the RKI[2–5], the B.1.617.2 variant (the one that caused most infections in summer/fall 2021) was first detected in Germany in Week 12 of 2021. In Weeks 14-15, only 8 such cases were confirmed[5]. (**A**) shows the (likely) distribution of the Delta variant across counties in Week 17 of 2021. Note that (**A**) covers both B.1.617.1 and B.1.617.2 (the RKI did not distinguish between the two subvariants at the county level in the corresponding report). (**B**) shows the counties where B.1.617.2 was confirmed or suspected in Weeks 18-20 of 2021. Cyan indicates counties where the virus has been detected in Week 17 but no longer afterwards. These counties either had the B.1.617.1 variant in Week 17, or no evidence was found that the B.1.617.2 variant had spread further in these counties in the following weeks. The dark blue ones are those with possible B.1.617.2 cases in both periods, Week 17 and Weeks 18-20. Green represents the counties with new cases in Weeks 18-20 that are adjacent to an infected county in Week 17. Yellow are infected counties that are adjacent to a county that is adjacent to an infected county in Week 17. Red are other infected counties. Red counties in particular indicate that the virus has spread over long distances. The colors in (**C**) are set accordingly for Week 21. The only difference is that we do not use cyan in (**C**) anymore; instead, white counties represent those with no new cases in Week 21, but some either in Week 17 or in the period 18-20.

Supplementary Discussion 2

Spread of the seasonal Flu. To complement our observations, we also investigated the seasonal flu. We again obtained the weekly incidence numbers per county in Germany from the RKI[7]. On their website a yearly report is released that summarizes the reported infection numbers for a variety of diseases, including the flu. Using the same methodology as in the case of COVID-19, we create a heatmap of outbreaks. We focus on a time frame of December 2019 to January 2020 at which a big wave of the flu hit Germany. More precisely, **Supplementary Fig. 2** shows how the flu spreads in the continuous four weeks starting from 12/30/2019 to 01/26/2020. In the 4 weeks before Week 1 (i.e., 4 weeks before 12/30/2019), the incidence number (per 100,000 inhabitants) averaged over all counties decreases from 50 to below 10. An increasing trend in incidence numbers then starts with Week 1, causing the average to rise above 29 in Week 4. This trend continues until the flu hits its seasonal high in Week 9 with an averaged incidence of 150 (not visible in **Supplementary Fig. 2**). Note, the incidence numbers only reflect the number of reported cases, normalized by the population size of the county. The real incidence numbers may be larger due to unreported cases. However, the data set for 2019 alone still records more than 190,000 cases of the flu, which were confirmed via laboratory tests[7]. Laboratories, doctors and hospitals are required to report positive tests for various diseases (including the flu) to the German ministry of health, which forwards the data to the RKI.

In **Supplementary Fig. 2** we observe a combination of the non-local spread observed in the context of COVID-19 with an increased spread to locally adjacent areas. In Week 2 many areas that are adjacent to areas highlighted in Week 1 experience an outbreak. An explanation for this spreading behavior which is different to COVID-19, could be the lack of countermeasures (i.e., mandatory FFP2 masks and home office). We suspect that this increases the relative impact of short- and medium-range travel on the spread of the disease. These transport modes include public commuting transport, which tends to be crowded and facilitates the spread of infections between individuals in adjacent counties. Additionally, we observe that the number counties that are highlighted increases quicker: in Week 3 roughly half of all counties are colored. Intuitively, this could also be explained by the lack of countermeasures.

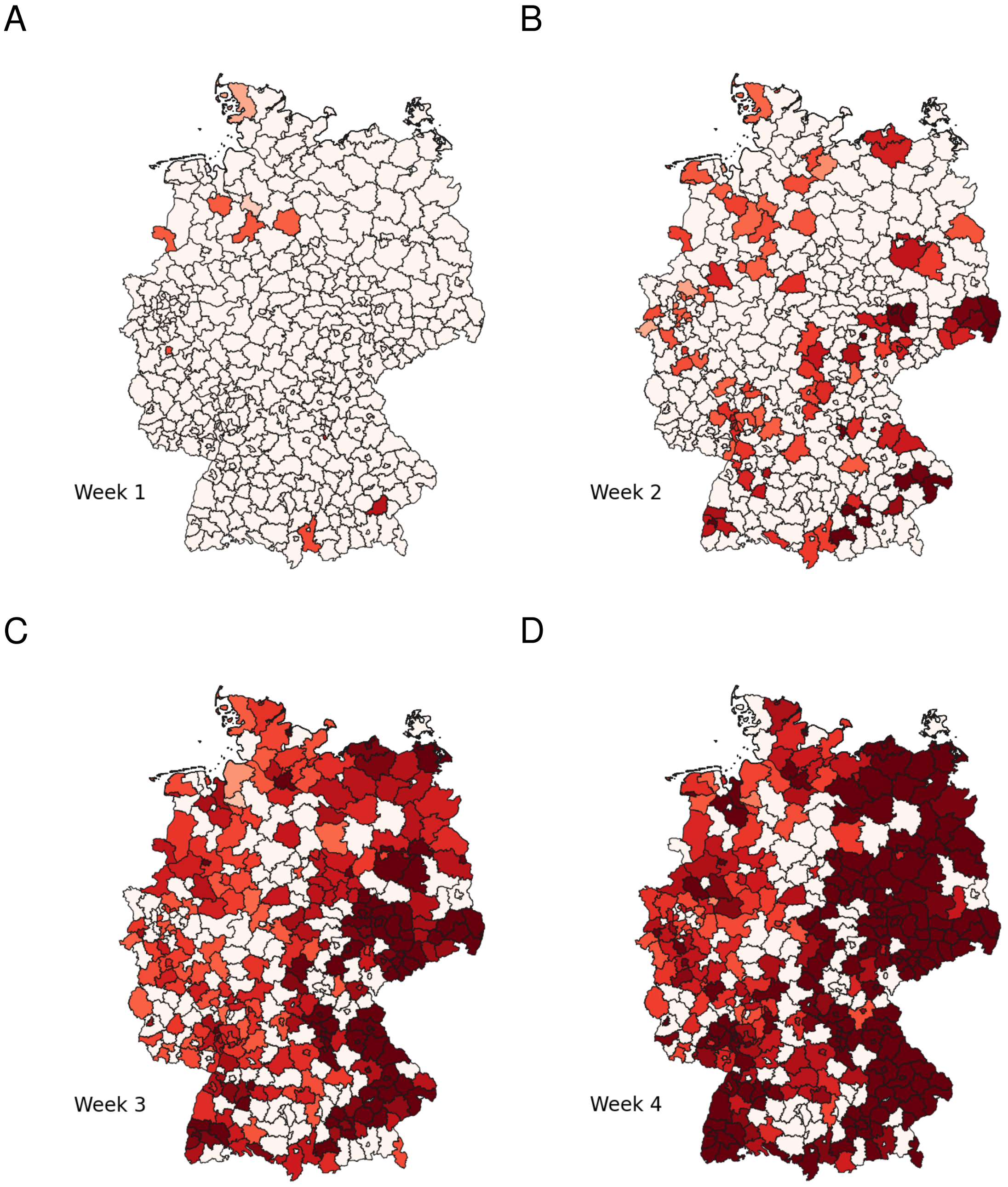

Supplementary Figure 2: The flu in Germany features both local and non-local spreading characteristics. The figure depicts the weekly influence incidences across all counties, following the same methodology for highlighting as applied in **Fig. 1** of the main paper. (**A**) shows the period from 12/30/2019 to 01/05/2020, (**B**) from 01/06/2020 to 01/12/2020, (**C**) from 01/13/2020 to 01/19/2020, and **d** from 01/20/2020 to 01/26/2020. In Week 1 the average of all incidence numbers lies below 8, increasing to above 29 in Week 4. Starting from Week 2 we observe that counties distant to the initially colored counties in Week 1, become highlighted as in our analysis of COVID-19 in **Fig. 1**. In addition, we observe a more local spreading pattern: some counties adjacent to the initial counties are colored in Week 2,3 and 4. The total number of colored counties increases faster than observed in our COVID-19 example.

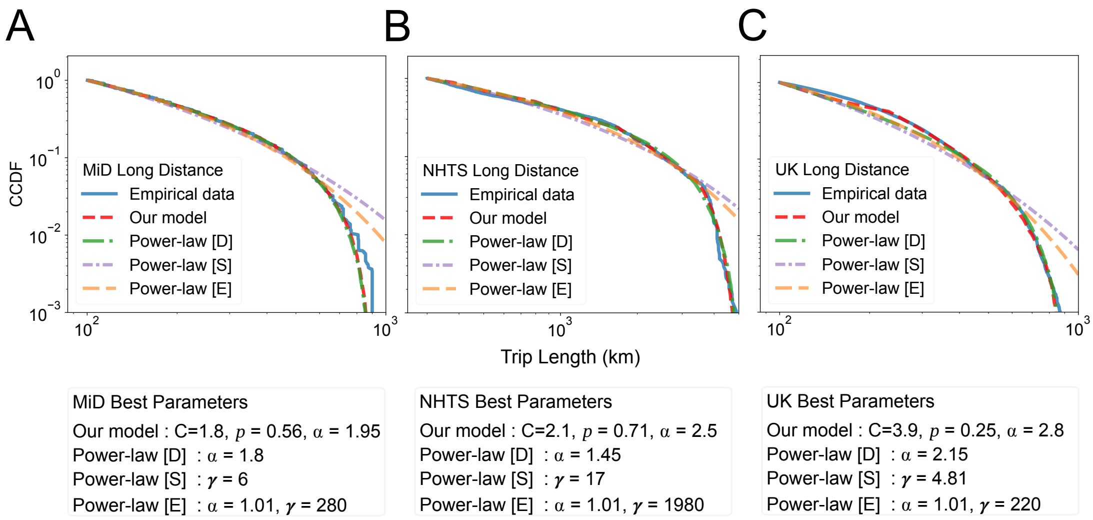

Supplementary Figure 3: Stretched exponential. We compare the stretched exponential to the models covered in our main paper in the context of the German MiD, U.S. NHTS and U.K. MNO data sets in (**A**), (**B**) and (**C**), respectively. Power-law [S] (colored in lavender) denotes the stretched-exponential[8] with density $f(x) \propto x^{-3/4} \cdot \exp(-x^{1/2}/\gamma)$ for a parameter $\gamma > 0$. It arises as a simplified form of a more complex acceleration-based model[8], which in its full formulation would rely on temporal data for training. The stretched exponential resembles a power law with fixed exponent α while the exponential cutoff is softened by a square-root dependence on the trip distance. We used grid search in combination with sMAPE to optimize the parameters for all displayed models (see Methodology section in the main paper). In all three experiments, the stretched exponential model is outperformed by the other considered models and has sMAPE values of 0.237, 0.416, and 0.427 in the MiD, NHTS and MNO experiments, respectively.

Supplementary References

[1] Brockmann, D. & Helbing, D. The hidden geometry of complex, network-driven contagion phenomena. *Science* **342**, 1337–1342 (2013).

[2] Robert Koch Insitut (RKI). Bericht zu Virusvarianten von SARS-CoV-2 in Deutschland - 26. Mai 2021 URL `https://www.rki.de/DE/Themen/Infektionskrankheiten/Infektionskrankheiten-A-Z/C/COVID-19-Pandemie/DESH/Berichte-VOC-tab.html`.

[3] Robert Koch Insitut (RKI). Bericht zu Virusvarianten von SARS-CoV-2 in Deutschland - 02. Juni 2021 URL `https://www.rki.de/DE/Themen/Infektionskrankheiten/Infektionskrankheiten-A-Z/C/COVID-19-Pandemie/DESH/Berichte-VOC-tab.html`.

[4] Robert Koch Insitut (RKI). Bericht zu Virusvarianten von SARS-CoV-2 in Deutschland - 09. Juni 2021 URL `https://www.rki.de/DE/Themen/Infektionskrankheiten/Infektionskrankheiten-A-Z/C/COVID-19-Pandemie/DESH/Berichte-VOC-tab.html`.

[5] Robert Koch Insitut (RKI). Bericht zu Virusvarianten von SARS-CoV-2 in Deutschland - 16. Juni 2021 URL `https://www.rki.de/DE/Themen/Infektionskrankheiten/Infektionskrankheiten-A-Z/C/COVID-19-Pandemie/DESH/Berichte-VOC-tab.html`.

[6] Robert Koch Insitut (RKI). Bericht zu Virusvarianten von SARS-CoV-2 in Deutschland - 23. Juni 2021 URL `https://www.rki.de/DE/Themen/Infektionskrankheiten/Infektionskrankheiten-A-Z/C/COVID-19-Pandemie/DESH/Berichte-VOC-tab.html`.

[7] Robert Koch Institute (RKI). Influenza incidence numbers per county in Germany. URL `https://survstat.rki.de/Content/Query/Create.aspx`. Further information can be found at `https://survstat.rki.de/Content/Instruction/Content.aspx`.

[8] Gallotti, R., Bazzani, A., Rambaldi, S. & Barthelemy, M. A stochastic model of randomly accelerated walkers for human mobility. *Nature Communications* **7** (2016). URL `https://www.nature.com/articles/ncomms12600`.
